# Cluster designs to assess the prevalence of acute malnutrition by lot quality assurance sampling: a validation study by computer simulation

**DOI:** 10.1111/j.1467-985X.2008.00572.x

**Published:** 2009-04

**Authors:** Casey Olives, Marcello Pagano, Megan Deitchler, Bethany L Hedt, Kari Egge, Joseph J Valadez

**Affiliations:** Harvard School of Public HealthBoston, USA; Academy for Educational DevelopmentWashington DC, USA; Harvard School of Public HealthBoston, USA; American Red CrossBangkok, Thailand; Liverpool School of Tropical MedicineUK

**Keywords:** Acute malnutrition, Emergency, Lot quality assurance sampling, Sequential sampling, Wasting

## Abstract

Traditional lot quality assurance sampling (LQAS) methods require simple random sampling to guarantee valid results. However, cluster sampling has been proposed to reduce the number of random starting points. This study uses simulations to examine the classification error of two such designs, a 67×3 (67 clusters of three observations) and a 33×6 (33 clusters of six observations) sampling scheme to assess the prevalence of global acute malnutrition (GAM). Further, we explore the use of a 67×3 sequential sampling scheme for LQAS classification of GAM prevalence. Results indicate that, for independent clusters with moderate intracluster correlation for the GAM outcome, the three sampling designs maintain approximate validity for LQAS analysis. Sequential sampling can substantially reduce the average sample size that is required for data collection. The presence of intercluster correlation can impact dramatically the classification error that is associated with LQAS analysis.

## 1. Introduction

In the last 20 years, development organizations working in international health have increasingly adopted lot quality assurance sampling (LQAS) to assess health care parameters. Nearly all of the 805 studies that were identified in a recent review of LQAS implemented between January 1984 and December 2004 employed traditional LQAS sampling methods (Robertson, 2006), in which simple random sampling (SRS) is used for data collection. The exceptions are studies in which a two-stage LQAS design was combined with cluster sampling to assess neonatal tetanus eradication (World Health Organization, 2001, 2002, 2004), and a study in which small clusters instead of SRS were used to assess the prevalence of gobal acute malnutrition (GAM) by LQAS analysis methods (Deitchler *et al.*, 2007).

In the international health setting, small sample sizes (e.g. *n*=19) have often been used for LQAS assessment of service provision indicators (Valadez, *et al.* 2003). The small samples sizes have meant that LQAS has been feasible for use by local managers (Valadez, 1991). However, use of LQAS for assessment of anthropometric indicators requires large sample sizes due to the increased precision that is needed for hypothesis testing. To use SRS with large sample sizes means an increase in time and cost, as data collection for each observation in the sample can require travel to a different site. Sampling observations in batches, or clusters, is an alternative method which reduces the number of site visits that are needed to complete data collection. However, if the observations within each cluster are highly correlated with respect to the outcome being assessed, cluster sampling leads to increased misclassification with the LQAS analysis method. In contrast, cluster sampling could be a viable option if it does not undermine the validity of the independence assumption for hypothesis testing, as required by LQAS.

Deitchler *et al.* (2007, 2008) field tested both a 67×3 and a 33×6 cluster design (67 clusters of size 3 and 33 clusters of size 6 respectively) for LQAS assessment of GAM prevalence in the Siraro woreda of Ethiopia in 2003 and in the administrative units of Fur Baranga and Habila in West Darfur in 2005. The use of a 67×3 sequential sampling design was also investigated in the Ethiopia study. In comparison with the 67×3 and 33×6 design, the sequential design allowed for a reduction in the total sample size that was required to assess the prevalence of GAM by LQAS analysis methods (Deitchler *et al.*, 2007). Similar sequential designs have been used for categorizing resistance of human immunodeficiency virus to drugs (Bennett *et al.*, 2006). However, those designs relied on SRS for validity.

The current study uses computer simulations to assess the validity of the small cluster approach that was used to assess the prevalence of GAM. The principal sampling strategy uses a cluster model to minimize the number of random sites to visit. We focus on a 67×3 and a 33×6 cluster design as these were the designs that were tested in Ethiopia and Sudan. Additionally, we develop and investigate a second strategy which applies a sequential sampling scheme to the 67×3 cluster design. Here, we use more robust statistical assumptions for the sequential design than had been applied to the work in Ethiopia, to improve the design.

## 2. Methods

### 2.1. Traditional lot quality assurance sampling methods

LQAS inference uses the binomial approximation to the hypergeometric distribution to test whether the prevalence of a parameter of interest is exhibited at a proportion that is greater than or equal to some prespecified threshold *P*_0_. This is equivalent to the hypothesis test 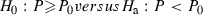 where *P* is the true prevalence in the population and *P*_0_, the upper threshold, is the prevalence level that the data are tested against. In the case of GAM, *P*_0_ represents an unacceptable level of acute malnutrition in the population. It is chosen to reflect the prevalence at which a population would be considered a priority for humanitarian intervention. The null hypothesis is rejected if the number of individuals in the sample exhibiting acute malnutrition, *s*, is less than or equal to an *a priori* defined critical value *d* (*s*≤*d*). This critical value is often referred to as the decision rule in LQAS literature (Valadez, 1991). In addition, LQAS requires that we define a lower threshold *P*_a_. The lower threshold reflects the prevalence of GAM at which the population would not be considered a priority intervention.

As with any hypothesis test, an *α*- and *β*-error are associated with LQAS. The *α*-error is the highest probability that the null hypothesis is incorrectly rejected. In the case of GAM, this would mean concluding that the assessment area does not have a high level of acute malnutrition when in fact it does. This probability is controlled for at the upper threshold: 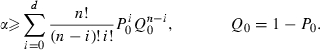


The *β*-error is the highest probability that we incorrectly fail to reject the null hypothesis. This would mean concluding that the assessment area does have a high level of acute malnutrition when in fact it does not. The *β*-error is controlled for at the lower threshold: 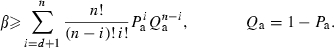


The critical value is chosen to approximate the desired *α* and *β* given the upper and lower thresholds, and the sample size. In practice, it is difficult to attain the *α*- and *β*-errors exactly owing to the discrete nature of the binomial distribution. Further, more than one critical value can achieve the specified constraints. The actual error probabilities for a specific sample size, and upper and lower thresholds, therefore depend on the critical value *d* that is chosen.

In this study, we investigate the upper and lower thresholds that were field tested in Ethiopia and Sudan (Deitchler *et al.*, 2007, 2008). Three couplets (i.e. upper–lower threshold pairs) are investigated: the upper thresholds of 10%, 15% and 20%, and the respective lower thresholds of 5%, 10% and 15%. The 10%–5% and 15%–10% couplet are of primary concern as these are the benchmarks that are most commonly used by humanitarian agencies to assess the severity of GAM prevalence (Food and Agriculture Organization and Food Security Analysis Unit, 2006). The 20%–15% couplet is of secondary consideration as prevalences of GAM above 20% are fairly rare, even in emergency settings (Médecins sans Frontières, 1995).

For each upper and lower threshold couplet, we determined the critical value subject to the constraints of an *α*-error of approximately 0.10 and a *β*-error of approximately 0.20 for samples

of sizes 198 (33×6) and 201 (67×3). Table 1 gives the sample size, critical value and associated *α*- and *β*-errors for each upper and lower threshold couplet when traditional SRS is used for data collection. For the 10%–5% couplet, a critical value of 13 meets the constraints of *α*≤0.10 and *β*≤0.20 for both sample sizes. For the 15%–10% couplet, the desired error limits are approximately maintained for a critical value of 23. For the 20%–15% couplet, no critical value attains or closely approximates the desired *α*- and *β*-constraints for samples of size 198 and 201. The critical value 33 minimizes the total error for a sample of size 198 and the critical value 34 minimizes the total error for a sample of size 201. We chose to use the critical value 33 for this couplet, with a corresponding *α* of 0.138 and *β* of 0.221.

**Table 1:** LQAS *α*- and *β*-errors, and critical values for samples sizes 198 and 201 for three upper and lower threshold couplets assuming SRS

*Sample size*	*Results for the following threshold pairs:*								
	*10%–5%*			*15%–10%*			*20%–15%*		
	*d*	*α*	*β*	*d*	*α*	*β*	*d*	*α*	*β*
201	12	0.031	0.208	22	0.061	0.279	32	0.085	0.315
	13	0.054	0.134	23	0.091	0.209	33	0.117	0.250
	14	0.089	0.081	24	0.131	0.151	34	0.157	0.193
198	12	0.035	0.194	22	0.072	0.255	32	0.101	0.283
	13	0.062	0.123	23	0.106	0.188	33	0.138	0.221
	14	0.101	0.073	24	0.150	0.134	34	0.183	0.169

### 2.2. Lot quality assurance sampling methods for sequential cluster designs

In this section we investigate a sequential cluster design to test the same three null hypotheses as above. The sequential cluster design differs from traditional LQAS as a decision can be made to reject or accept the null hypothesis after each individual cluster has been observed. In a *k*×*m* sequential sampling design, there are at most *k* stages of sampling. At each stage, *m* sampling elements are observed for a maximum of *n* possible observations. At the *i*th stage of sampling, we define a rejection rule *r*_*i*_, an acceptance rule *a*_*i*_ and the cumulative number of outcomes, *s*_*i*_ (in our application, an outcome is a child exhibiting GAM). If *s*_*i*_≥*a*_*i*_, then we conclude that the prevalence of GAM is greater than or equal to *P*_0_, and sampling stops. Likewise, if *s*_*i*_≤*r*_*i*_, then we conclude that the prevalence is less than *P*_0_, and sampling stops. Otherwise, if *r*_*i*_<*s*_*i*_<*a*_*i*_, sampling proceeds to the next stage. If no decision is made by the time that the final (*k*th) stage of sampling is reached, then a decision is made to reject if *s*_*n*_≤(*a*_*n*_+*r*_*n*_)/2 and to accept if *s*_*n*_>(*a*_*n*_+*r*_*n*_)/2.

Wald outlined the calculation of LQAS critical values at each stage of a sequential design applied to observations that are selected by SRS (Wald, 1947). These critical values are linear in the individual observations. We adapt this theory to accommodate clusters of size *m* (*m*>1), under the assumption that observations within each cluster are independent. Namely, define 
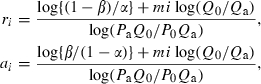
 where *α* and *β* refer to the target classification errors. These critical values are linear in the sampling stage and thus reflect a cluster sampling design.

One of the benefits of sequential designs is the potential for reduction of the overall sample size that is required for data collection. With respect to the outcome of acute malnutrition, a reduction in sample size could lead to a more rapid response to an emergency situation. The average sample number ASN, or the average number of clusters that are sampled to reject or accept the null hypothesis, characterizes this reduction. The average sample size is equal to the number of sampling elements per cluster times ASN (*m* ASN) and is given by the formula 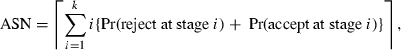 where *f*(*x*)=⌈(·)⌉ is the next largest integer function (Aroian, 1976).

The Wald critical values rely on the assumption that the number of possible observations is unbounded. However, in virtually all applications, this is not so. When the number of possible observations is bounded, the design is said to be truncated. The use of Wald critical values in truncated sequential designs does not generally yield the appropriate *α* and *β* (Wald, 1947). Aroian (1965, 1976) suggested treating a sequential sample as a random walk to calculate the classification error for a truncated design directly. We used Aroian's direct method to calculate the true classification error for a range of sequential designs varied over the parameter space of *α* and *β* to arrive within the desired targets of classification error.

Here we investigate a 67×3 sequential sampling design with application to the three upper–lower threshold couplets of interest. In terms of the above notation, *k*=67, *m*=3, *n*=201 and the upper bound for ASN is 67. For each upper and lower threshold couplet, we determine the acceptance and rejection rules by using Wald theory. We calculated critical values for a range of *α*- and *β*-errors around the target levels of 0.10 and 0.20 respectively. The final critical values that are chosen are those that yield the true *α* and *β* nearest to the desired levels as calculated by using the direct method. For both the 15%–10% and 20%–15% couplets, we could not find a design that yielded the desired *α*- and *β*-targets. For these couplets we selected the design that jointly minimized the *α*- and *β*-errors. For the 10%–5% couplet we expect an *α* of 0.10 and a *β* of 0.16. For the 15%–10% couplet, we expect an *α* of 0.10 and a *β* of 0.24. And, for the 20%–15% couplet, we expect an *α* of 0.17 and a *β* of 0.22. The critical values for each couplet are given in Table 2.

**Table 2:** Rejection (*r*) and acceptance (*a*) rules for the 67×3 sequential design for three upper and lower threshold couplets assuming complete independence†

*Stage*	*Results for the following couplets* (*P*_0_/*P*_a_):					
	*10%–5%*		*15%–10%*		*20%–15%*	
	*r*	*a*	*r*	*a*	*r*	*a*
1	ND	3	ND	4	ND	5
2	ND	3	ND	5	ND	6
3	ND	3	ND	5	ND	7
4	ND	3	ND	5	ND	7
5	ND	4	ND	6	ND	8
6	ND	4	ND	6	ND	8
7	ND	4	ND	6	ND	9
8	ND	4	ND	7	ND	9
9	ND	4	ND	7	ND	10
10	ND	5	ND	8	ND	10
11	ND	5	ND	8	ND	11
12	ND	5	ND	8	0	11
13	ND	5	ND	9	0	12
14	ND	6	ND	9	1	12
15	0	6	ND	9	1	13
16	0	6	ND	10	2	13
17	0	6	0	10	2	14
18	0	6	0	11	3	14
19	1	7	1	11	3	15
20	1	7	1	11	4	15
21	1	7	1	12	4	16
22	1	7	2	12	5	16
23	1	8	2	12	6	17
24	2	8	2	13	6	17
25	2	8	3	13	7	18
26	2	8	3	14	7	19
27	2	8	4	14	8	19
28	2	9	4	14	8	20
29	3	9	4	15	9	20
30	3	9	5	15	9	21
31	3	9	5	15	10	21
32	3	9	5	16	10	22
33	4	10	6	16	11	22
34	4	10	6	16	11	23
35	4	10	6	17	12	23
36	4	10	7	17	12	24
37	4	11	7	18	13	24
38	5	11	8	18	13	25
39	5	11	8	18	14	25
40	5	11	8	19	14	26
41	5	11	9	19	15	26
42	6	12	9	19	15	27
43	6	12	9	20	16	27
44	6	12	10	20	16	28
45	6	12	10	21	17	28
46	6	13	11	21	18	29
47	7	13	11	21	18	30
48	7	13	11	22	19	30
49	7	13	12	22	19	31
50	7	13	12	22	20	31
51	7	14	12	23	20	32
52	8	14	13	23	21	32
53	8	14	13	24	21	33
54	8	14	14	24	22	33
55	8	14	14	24	22	34
56	9	15	14	25	23	34
57	9	15	15	25	23	35
58	9	15	15	25	24	35
59	9	15	15	26	24	36
60	9	16	16	26	25	36
61	10	16	16	26	25	37
62	10	16	16	27	26	37
63	10	16	17	27	26	38
64	10	16	17	28	27	38
65	11	17	18	28	27	39
66	11	17	18	28	28	39
67	14	15	23	24	34	35

† ND signifies that no decision is made and sampling continues.

### 2.3. Simulation validation of cluster designs for lot quality assurance sampling analysis

One key assumption in LQAS theory is that SRS is used for data collection of binary outcomes (Hoshaw-Woodard, 2001; Valadez, 1991). Cluster sampling often results in an intracluster correlation (correlation between subjects within the same cluster with respect to the outcome of interest). For the cluster designs that are of concern here, intracluster correlation could result from within-household correlation (i.e. correlation of GAM between multiple children sampled in one household) or as correlation of GAM between multiple households sampled within the same cluster (Deitchler *et al.*, 2007). Intercluster correlation (correlation between subjects in different clusters) is also possible although this is likely to be minimal for acute malnutrition and can be assumed to be less than or equal to the intracluster correlation (Fenn *et al.*, 2004; Reed, 2000). Validation of the 67×3,33×6 and sequential cluster design requires assessing the effect of these potential correlations on the *α*- and *β*-errors that are associated with LQAS hypothesis testing.

For the cluster sampling techniques that are investigated here, we assume that intracluster correlation is homogeneous and non-negative. Intercluster correlation is also assumed to be homogeneous and non-negative, and less than or equal to the intracluster correlation. This study confines the investigation to the intercluster and intracluster correlations of 0.00, 0.05, 0.10, 0.15, 0.20 and 0.25, because these provide a broad set of acceptable alternatives. Kalton's work on cluster sampling suggests that intracluster correlation is usually less than 0.15 for most indicators (Kalton, 1983). The well-documented multiple causes of malnutrition along with the age dependence vulnerability of children to acute malnutrition (Shrimpton *et al.*, 2001; United Nations Children's Fund, 1990) further suggest that a low intracluster correlation is likely. Moreover, a review of demographic and health surveys that were conducted in 46 developing countries reported intracluster correlations of less than 0.10 for acute malnutrition in 90% of the countries that were studied (Fenn *et al.*, 2004) and intracluster correlations of less than 0.05 were reported for GAM in field applications of the 67×3 and 33×6 designs in Sudan (Deitchler *et al.*, 2008). With these considerations in mind, we expect intracluster correlations using the three cluster sampling schemes used here to be less than 0.05 in most field settings. Intracluster correlation levels equal to and above 0.05 for GAM, although unlikely, are investigated in this study to understand the effect of unusually high levels of intracluster correlation on LQAS classification error for these designs.

### 2.4. Simulation methods

To reproduce the correlation structure arising from the 67×3 and 33×6 sampling schemes and the 67×3 sequential sampling scheme, it is necessary to generate correlated binary vectors **D** such that **D**∼(**P**,**Σ**) where **P** is the *n*×1 mean vector of *P*s and **Σ** is the *n*×*n* variance–covariance matrix describing the correlation structure. For each couplet, samples of size 201 and 198 were generated under the various intercluster and intracluster correlation constraints. This procedure was repeated 10000 times for each couplet and intercluster–intracluster correlation pair for each design. All simulations were performed by using the statistical package R version 2.6.0 (R Development Core Team, 2007). The simulation methodology is described in detail in Appendix A.

## 3. Results

### 3.1. Cluster sampling strategy: the 67×3 and 33×6 designs

Tables 3–5 contain the results of the simulations for the 67×3 and 33×6 designs along with the estimated standard errors. As expected, those simulations with an intercluster and intracluster correlation equal to 0 for GAM demonstrate *α*- and *β*-errors that are approximately equal to the binomial *α*- and *β*-errors that are presented in Table 1, as this situation corresponds to SRS.

**Table 3:** Simulation results for the 67×3 and 33×6 designs: *α*- and *β*-errors for the 10%–5% couplet with varied intercluster and intracluster correlation and *d*= 13†

*Correlation*		*Results for the 67*×*3 design*		*Results for the 33*×*6 design*	
*Intercluster*	*Intracluster*	*α*	*β*	*α*	*β*
0.00	0.00	0.054 (0.002)	0.136 (0.003)	0.061 (0.002)	0.124 (0.003)
0.00	0.05	0.064 (0.002)	0.144 (0.004)	0.083 (0.003)	0.147 (0.004)
0.05		0.389 (0.005)	0.247 (0.004)	0.402 (0.005)	0.253 (0.004)
0.00	0.10	0.071 (0.003)	0.159 (0.004)	0.103 (0.003)	0.161 (0.004)
0.05		0.390 (0.005)	0.263 (0.004)	0.393 (0.005)	0.246 (0.004)
0.10		0.491 (0.005)	0.234 (0.004)	0.488 (0.005)	0.245 (0.004)
0.00	0.15	0.077 (0.003)	0.162 (0.004)	0.123 (0.003)	0.188 (0.004)
0.05		0.389 (0.005)	0.246 (0.004)	0.394 (0.005)	0.246 (0.004)
0.10		0.473 (0.005)	0.239 (0.004)	0.495 (0.005)	0.237 (0.004)
0.15		0.552 (0.005)	0.220 (0.004)	0.551 (0.005)	0.216 (0.004)
0.00	0.20	0.086 (0.003)	0.172 (0.004)	0.141 (0.003)	0.197 (0.004)
0.05		0.389 (0.005)	0.258 (0.004)	0.407 (0.005)	0.245 (0.004)
0.10		0.487 (0.005)	0.236 (0.004)	0.491 (0.005)	0.231 (0.004)
0.15		0.550 (0.005)	0.230 (0.004)	0.550 (0.005)	0.221 (0.004)
0.20		0.592 (0.005)	0.208 (0.004)	0.599 (0.005)	0.205 (0.004)
0.00	0.25	0.097 (0.003)	0.179 (0.004)	0.163 (0.004)	0.205 (0.004)
0.05		0.400 (0.005)	0.256 (0.004)	0.409 (0.005)	0.255 (0.004)
0.10		0.478 (0.005)	0.236 (0.004)	0.491 (0.005)	0.239 (0.004)
0.15		0.552 (0.005)	0.225 (0.004)	0.548 (0.005)	0.215 (0.004)
0.20		0.592 (0.005)	0.213 (0.004)	0.595 (0.005)	0.198 (0.004)
0.25		0.628 (0.005)	0.195 (0.004)	0.635 (0.005)	0.187 (0.004)

† Standard errors are given in parentheses.

In the correlated samples, the least effect on *α*- and *β*-error occurs when the intercluster correlation equals 0. For example, in the case of the 67×3 design, if the intercluster correlation is equal to 0 and the intracluster correlation is less than or equal to 0.25, the 10%–5% couplet maintains the desired error limits of *α*≤0.10 and *β*≤0.20 (Table 3). With intracluster correlations less than 0.10 the 15%–10% couplet performs approximately within the desired error limits (Table 4). Although the 20%–15% couplet has errors that are slightly above the desired limits at this correlation level, these were expected from the outset as the targets were untenable under SRS (Table 5).

**Table 5:** Simulation results for the 67×3 and 33×6 designs: *α*- and *β*-errors for the 20%–15% couplet with varied intercluster and intracluster correlation and *d*=33†

*Correlation*		*Results for the 67*×*3 design*		*Results for the 33*×*6 design*	
*Intercluster*	*Intracluster*	*α*	*β*	*α*	*β*
0.00	0.00	0.118 (0.003)	0.248 (0.004)	0.135 (0.003)	0.227 (0.004)
0.00	0.05	0.129 (0.003)	0.256 (0.004)	0.165 (0.004)	0.244 (0.004)
0.05		0.401 (0.005)	0.361 (0.005)	0.423 (0.005)	0.342 (0.005)
0.00	0.10	0.138 (0.003)	0.266 (0.004)	0.190 (0.004)	0.262 (0.004)
0.05		0.409 (0.005)	0.357 (0.005)	0.422 (0.005)	0.343 (0.005)
0.10		0.475 (0.005)	0.358 (0.005)	0.481 (0.005)	0.339 (0.005)
0.00	0.15	0.154 (0.004)	0.276 (0.004)	0.210 (0.004)	0.281 (0.004)
0.05		0.415 (0.005)	0.366 (0.005)	0.425 (0.005)	0.344 (0.005)
0.10		0.474 (0.005)	0.350 (0.005)	0.487 (0.005)	0.338 (0.005)
0.15		0.510 (0.005)	0.339 (0.005)	0.525 (0.005)	0.336 (0.005)
0.00	0.20	0.159 (0.004)	0.274 (0.004)	0.223 (0.004)	0.283 (0.005)
0.05		0.418 (0.005)	0.364 (0.005)	0.421 (0.005)	0.352 (0.005)
0.10		0.481 (0.005)	0.348 (0.005)	0.484 (0.005)	0.334 (0.005)
0.15		0.511 (0.005)	0.328 (0.005)	0.527 (0.005)	0.334 (0.005)
0.20		0.547 (0.005)	0.329 (0.005)	0.544 (0.005)	0.323 (0.005)
0.00	0.25	0.168 (0.004)	0.282 (0.004)	0.239 (0.004)	0.291 (0.005)
0.05		0.408 (0.005)	0.365 (0.005)	0.435 (0.005)	0.353 (0.005)
0.10		0.481 (0.005)	0.353 (0.005)	0.477 (0.005)	0.353 (0.005)
0.15		0.511 (0.005)	0.340 (0.005)	0.523 (0.005)	0.337 (0.005)
0.20		0.540 (0.005)	0.335 (0.005)	0.553 (0.005)	0.322 (0.005)
0.25		0.561 (0.005)	0.314 (0.005)	0.574 (0.005)	0.313 (0.005)

† Standard errors are given in parentheses.

**Table 4:** Simulation results for the 67×3 and 33×6 designs: *α*- and *β*-errors for the 15%–10% couplet with varied intercluster and intracluster correlation and *d*= 23†

*Correlation*		*Results for the 67*×*3 design*		*Results for the 33*×*6 design*	
*Intercluster*	*Intracluster*	*α*	*β*	*α*	*β*
0.00	0.00	0.095 (0.003)	0.211 (0.004)	0.107 (0.003)	0.185 (0.004)
0.00	0.05	0.096 (0.003)	0.215 (0.004)	0.137 (0.003)	0.210 (0.004)
0.05		0.407 (0.005)	0.317 (0.005)	0.420 (0.005)	0.310 (0.005)
0.00	0.10	0.111 (0.003)	0.220 (0.004)	0.155 (0.004)	0.230 (0.004)
0.05		0.410 (0.005)	0.324 (0.005)	0.426 (0.005)	0.314 (0.005)
0.10		0.479 (0.005)	0.312 (0.005)	0.488 (0.005)	0.304 (0.005)
0.00	0.15	0.115 (0.003)	0.230 (0.004)	0.173 (0.004)	0.241 (0.004)
0.05		0.393 (0.005)	0.327 (0.005)	0.418 (0.005)	0.317 (0.005)
0.10		0.489 (0.005)	0.315 (0.005)	0.497 (0.005)	0.307 (0.005)
0.15		0.538 (0.005)	0.301 (0.005)	0.525 (0.005)	0.288 (0.005)
0.00	0.20	0.135 (0.003)	0.248 (0.005)	0.192 (0.004)	0.264 (0.004)
0.05		0.407 (0.005)	0.319 (0.005)	0.415 (0.005)	0.316 (0.005)
0.10		0.485 (0.005)	0.308 (0.005)	0.491 (0.005)	0.298 (0.005)
0.15		0.527 (0.005)	0.305 (0.005)	0.537 (0.005)	0.292 (0.005)
0.20		0.562 (0.005)	0.283 (0.005)	0.577 (0.005)	0.279 (0.004)
0.00	0.25	0.138 (0.003)	0.247 (0.004)	0.205 (0.004)	0.270 (0.004)
0.05		0.403 (0.005)	0.328 (0.005)	0.425 (0.005)	0.322 (0.005)
0.10		0.481 (0.005)	0.304 (0.005)	0.488 (0.005)	0.306 (0.005)
0.15		0.525 (0.005)	0.301 (0.005)	0.536 (0.005)	0.290 (0.005)
0.20		0.557 (0.005)	0.285 (0.005)	0.575 (0.005)	0.279 (0.004)
0.25		0.594 (0.005)	0.278 (0.004)	0.597 (0.005)	0.261 (0.004)

† Standard errors are given in parentheses.

In the case of the 33×6 design, assuming an intercluster correlation equal to 0, the 10%–5% couplet conforms to the desired error limits of *α*≤0.10 and *β*≤0.20 for intracluster correlations up to 0.10 (Table 3); the 15%–10% couplet conforms approximately to the desired error limits when the intracluster correlation equals 0, and, as expected, the 20%–15% couplet does not attain the desired performance (Tables 4 and 5).

In cases where both the intercluster and the intracluster correlation are greater than 0, there is a substantial increase in the *α*-error for both the 67×3 and the 33×6 designs, though the *β*-error is less affected. This result suggests that, when intercluster correlation is greater than 0, larger samples may be required to attain the desired *α*- and *β*-levels. On use of random methods for selection of clusters to sample, it is, however, reasonable to assume an intercluster correlation equal to 0 for LQAS assessment of GAM prevalence with the 67×3 or 33×6 design.

### 3.2. Sequential sampling strategy: the 67×3 sequential design

Table 6 shows the simulation results for the 67×3 sequential design. As expected, when intercluster and intracluster correlations are equal to 0, the results closely approximate the *α*- and *β*-errors that were calculated under SRS. Additionally, the least effect on the *α*- and *β*-errors occurs in simulations where the intercluster correlation is equal to 0. Assuming an intercluster correlation equal to 0 and an intracluster correlation as high as 0.25, the *α*-error is 0.16 or less and the *β*-error is 0.25 or less for the 10%–5% couplet. For the 15%–10% couplet, the *α*- and *β*-errors are 0.14 or less and 0.30 or less respectively. The errors for the 20%–15% couplet are slightly higher with the *α*-error 0.211 or less and the *β*-error 0.284 or less.

**Table 6:** Simulation results for the 67×3 sequential design: *α*- and *β*-errors with ASN for three upper and lower threshold couplets with varied intercluster and intracluster correlation†

*Correlation*		*Results for the 10%–5% couplet*				*Results for the 15%–10% couplet*				*Results for the 20%–15% couplet*			
*Intercluster*	*Intracluster*	*α*	*β*	*ASN*_0_	*ASN*_a_	*α*	*β*	*ASN*_0_	*ASN*_a_	*α*	*β*	*ASN*_0_	*ASN*_a_
0.00	0.00	0.095	0.160	22.933	33.048	0.087	0.240	34.273	49.094	0.150	0.217	39.707	46.955
		(0.003)	(0.004)	(17.914)	(17.452)	(0.003)	(0.004)	(21.493)	(18.291)	(0.004)	(0.004)	(21.124)	(18.685)
0.00	0.05	0.103	0.178	22.567	31.543	0.096	0.259	34.015	47.494	0.162	0.235	38.436	45.529
		(0.003)	(0.004)	(17.808)	(17.186)	(0.003)	(0.004)	(21.773)	(18.828)	(0.004)	(0.004)	(21.057)	(19.059)
0.05		0.401	0.251	22.576	23.309	0.392	0.335	30.509	32.414	0.435	0.345	30.348	30.596
		(0.005)	(0.004)	(18.066)	(14.715)	(0.005)	(0.005)	(21.187)	(18.853)	(0.005)	(0.005)	(20.214)	(18.905)
0.00	0.10	0.125	0.197	22.519	30.109	0.107	0.268	33.95	46.158	0.182	0.247	37.939	44.01
		(0.003)	(0.004)	(17.856)	(16.807)	(0.003)	(0.004)	(21.782)	(19.059)	(0.004)	(0.004)	(21.241)	(19.359)
0.05		0.401	0.257	21.946	22.831	0.396	0.330	30.305	32.322	0.427	0.335	29.858	30.289
		(0.005)	(0.004)	(17.558)	(14.678)	(0.005)	(0.005)	(21.075)	(18.692)	(0.005)	(0.005)	(20.108)	(18.494)
0.10		0.486	0.245	20.37	20.75	0.479	0.308	27.638	28.16	0.485	0.345	25.809	25.617
		(0.005)	(0.004)	(16.199)	(13.411)	(0.005)	(0.005)	(19.953)	(17.646)	(0.005)	(0.005)	(18.87)	(17.821)
0.00	0.15	0.135	0.206	21.747	28.99	0.119	0.290	33.104	44.276	0.186	0.258	36.302	42.393
		(0.003)	(0.004)	(17.632)	(16.394)	(0.003)	(0.005)	(21.656)	(19.614)	(0.004)	(0.004)	(21.235)	(19.326)
0.05		0.394	0.257	21.381	22.3	0.398	0.329	29.672	31.612	0.429	0.344	29.245	29.591
		(0.005)	(0.004)	(17.219)	(14.059)	(0.005)	(0.005)	(20.891)	(18.447)	(0.005)	(0.005)	(19.832)	(18.446)
0.10		0.493	0.239	20.32	20.182	0.470	0.314	27.101	27.32	0.495	0.349	25.779	25.253
		(0.005)	(0.004)	(15.945)	(12.808)	(0.005)	(0.005)	(19.904)	(17.547)	(0.005)	(0.005)	(18.913)	(17.567)
0.15		0.561	0.227	19.201	19.046	0.517	0.296	25.143	25.399	0.527	0.340	23.265	23
		(0.005)	(0.004)	(15.149)	(12.248)	(0.005)	(0.005)	(18.754)	(16.745)	(0.005)	(0.005)	(18.173)	(16.886)
0.00	0.20	0.149	0.214	21.406	28.003	0.131	0.298	32.52	43.029	0.199	0.269	35.989	41.115
		(0.004)	(0.004)	(17.343)	(16.382)	(0.003)	(0.005)	(21.729)	(19.681)	(0.004)	(0.004)	(21.293)	(19.583)
0.05		0.396	0.259	20.641	21.945	0.397	0.343	28.926	30.674	0.425	0.350	28.693	29.18
		(0.005)	(0.004)	(16.63)	(13.911)	(0.005)	(0.005)	(20.722)	(18.336)	(0.005)	(0.005)	(19.632)	(18.41)
0.10		0.494	0.248	19.927	19.948	0.475	0.319	26.894	26.982	0.489	0.342	25.352	25.06
		(0.005)	(0.004)	(15.853)	(12.778)	(0.005)	(0.005)	(19.822)	(17.272)	(0.005)	(0.005)	(18.715)	(17.169)
0.15		0.553	0.225	19.04	18.989	0.516	0.304	24.959	24.75	0.519	0.327	22.867	22.871
		(0.005)	(0.004)	(15.084)	(12.159)	(0.005)	(0.005)	(18.712)	(16.283)	(0.005)	(0.005)	(17.792)	(16.67)
0.20		0.590	0.210	18.432	18.034	0.554	0.281	23.452	23.621	0.553	0.319	21.601	21.155
		(0.005)	(0.004)	(14.329)	(11.208)	(0.005)	(0.004)	(17.862)	(15.817)	(0.005)	(0.005)	(17.335)	(16.099)
0.00	0.25	0.158	0.243	20.872	26.806	0.140	0.300	31.899	41.276	0.211	0.284	34.936	39.666
		(0.004)	(0.004)	(16.861)	(15.91)	(0.003)	(0.005)	(21.734)	(19.831)	(0.004)	(0.005)	(21.099)	(19.669)
0.05		0.400	0.261	20.294	21.574	0.396	0.346	28.724	30.247	0.428	0.352	27.96	28.537
		(0.005)	(0.004)	(16.454)	(13.355)	(0.005)	(0.005)	(20.627)	(18.269)	(0.005)	(0.005)	(19.367)	(17.946)
0.10		0.479	0.247	19.054	19.646	0.469	0.320	26.375	27.095	0.479	0.351	24.808	24.777
		(0.005)	(0.004)	(15.346)	(12.419)	(0.005)	(0.005)	(19.544)	(17.22)	(0.005)	(0.005)	(18.412)	(17.161)
0.15		0.545	0.231	18.609	18.752	0.516	0.299	24.36	24.741	0.528	0.333	22.457	22.523
		(0.005)	(0.004)	(14.461)	(11.812)	(0.005)	(0.005)	(18.493)	(16.105)	(0.005)	(0.005)	(17.425)	(16.403)
0.20		0.593	0.208	18.097	17.829	0.557	0.287	23.245	23.094	0.546	0.322	20.935	20.758
		(0.005)	(0.004)	(14.004)	(10.931)	(0.005)	(0.005)	(17.661)	(15.263)	(0.005)	(0.005)	(16.909)	(15.829)
0.25		0.622	0.192	17.634	17.479	0.594	0.268	22.23	22.177	0.575	0.315	19.744	19.707
		(0.005)	(0.004)	(13.438)	(10.792)	(0.005)	(0.004)	(17.026)	(15.051)	(0.005)	(0.005)	(16.143)	(15.4)

† Standard errors are given in parentheses.

For all simulated sequential samples, ASN is substantially less than the maximum of 67. For the 10%–5% couplet, the maximum ASN is approximately 23 under the null hypothesis and 34 under the alternative (*n*=69 and *n*=102 respectively). For the 15%–10% couplet, the maximum ASN is approximately 35 under the null hypothesis and 50 under the alternative (*n*=105 and *n*=150 respectively) and, for the 20%–15% couplet, the maximum ASN is 40 under the null hypothesis and 47 under the alternative (*n*=120 and *n*=141 respectively). This result suggests that the 67×3 sequential design could be utilized to decrease the total number of clusters sampled, and thus the overall sample size that is required for data collection. A slightly elevated level of misclassification, beyond *α*≤0.10 and *β*≤0.20, would need to be acceptable for the 15%–10% and 20%–15% couplets but, in cases where uncorrelated clusters and a low intracluster correlation can be assumed for GAM, the design may be appropriate to use.

## 4. Discussion

This study uses computer simulations to assess three cluster sampling schemes that were field tested in Ethiopia to assess the prevalence of GAM by LQAS analysis methods (Deitchler *et al.*, 2007). The simulation results show that the 67×3 and 33×6 cluster designs conform to the desired error limits of *α*≤0.10 and *β*≤0.20 for the 10%–5% and 15%–10% couplet at numerous intracluster correlation levels when the intercluster correlation is equal to 0. It stands to reason that the 67×3 design conforms to the desired *α*- and *β*-limits at higher intracluster correlation levels than the 33×6 design for both the 10%–5% and 15%–10% couplet. For the 10%–5% couplet, the 67×3 design maintains the desired error limits when the intercluster correlation is 0 and the intracluster correlation is as high as 0.25. For the 15%–10% couplet, the 67×3 design maintains *α* and *β* approximately equal to 0.10 and 0.20 when the intercluster correlation is equal to 0 and the intracluster correlation is less than 0.10. Therefore, when clusters can be assumed independent and correlation within the clusters can be assumed to be less than 0.10, the 67×3 design can be an effective method to reduce the number of sites that would otherwise need to be visited by SRS of the same size. In cases where the clusters can be assumed independent and correlation within the clusters less than 0.15, the 33×6 design can also be an effective method for assessing the prevalence of GAM, allowing for LQAS inference within the desired error limits for the 10%–5% couplet. To maintain the same error limits for the 15%–10% couplet with the 33×6 design, there can be no intracluster correlation. Intuitively, we expect the 67×3 design to perform within the desired error limits at higher levels of intracluster correlation than the 33×6 design, as smaller clusters would suffer less from intracluster correlation.

The simulation results for the 67×3 sequential design indicate a potential time advantage over the 67×3 and 33×6 cluster designs because the total sample required for data collection is likely to be smaller. However, notwithstanding two exceptions, the simulation results indicate that the *α*- and *β*-errors for all intercluster and intracluster correlation levels, for each threshold couplet, exceed the desired *α*- and *β*-limits of 0.10 and 0.20 respectively. Use of the sequential design with these maximal sample sizes would therefore be recommended only when it is acceptable to deviate slightly from the above-stated limits of *α* and *β*.

The results of this simulation study demonstrate that information about the intracluster correlation of GAM is needed to use the 67×3, 33×6 and 67×3 sequential sampling designs reliably for LQAS assessment of the prevalence of GAM. The review of demographic and health surveys by Fenn *et al.* (2004) suggests that most field settings will have an acute malnutrition intracluster correlation of less than 0.10, whereas the field application of the 67×3 and 33×6 designs in Sudan of Deitchler *et al.* (2008) suggests that an intracluster correlation of less than 0.05 is likely. These studies provide useful information about the plausible upper limit of intracluster correlation for acute malnutrition. However, investigators rarely know in advance the exact intracluster correlation that exists in a field setting where a malnutrition assessment will be conducted. Until there is more clarity about the conditions in which the upper levels of 0.05–0.10 intracluster correlation of GAM would be expected, or possibly exceeded, investigators desiring strict adherence to the stated LQAS error limits of *α*≤0.10 and *β*≤0.20 may prefer to err on the side of caution by using the better performing 67×3 design, whereas investigators who require data rapidly may prefer instead to use the 67×3 sequential design. Finally, those investigators seeking a balance between limited classification error and potential expediency of data collection may find that the 33×6 design meets their data requirements best.

The results of this study support use of the cluster designs that were used in Ethiopia and Sudan (Deitchler *et al.* 2007, 2008) for detecting threshold levels of GAM prevalence by LQAS analysis methods. Further, the findings from this study provide useful information to investigators who need to decide which design (i.e. a 67×3, 33×6 or 67×3 sequential design) best suits their analytic needs, with respect to expediency of data collection, and desired limits of classification error. The cluster sampling schemes that were analysed here offer both time efficient and statistically valid alternatives to the conventional methodology for assessment of acute malnutrition in emergency settings.

## Figures and Tables

**Fig. 1:**
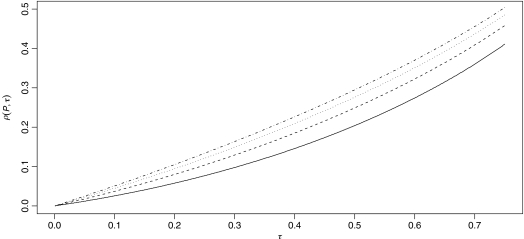
Approximation of the Bernoulli correlation as a function of bivariate normal correlation and prevalence: 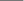, *P*=0.05; 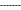, *P*=0.1; 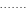, *P*=0.15; ·-·-·-, *P*=0.2

## Appendix A

This appendix details the methodology that was used for simulating binary random vectors with a correlation structure that reflects that which we might expect when applying the 67×3 and 33×6 cluster designs. There are few discrete probabilistic distributions which easily lend themselves to simulation of correlated binary observations. We outline a specific method for generating binary random vectors that is based on truncation of multivariate normal random variables.

### A.1. Simulation

For a *k*×*m* cluster sample (*k* is the number of clusters and *m* is the size of each cluster for a total sample of size *n*), it is necessary to generate clusters with specific intercluster and intracluster correlation subject to the constraint that the intercluster correlation is less than or equal to the intracluster correlation. Let ***τ***_1_=*τ*_1_**11**^T^+(1−*τ*_1_)**I** and ***τ***_2_=*τ*_2_**11**^T^, where **1** is an *m*×1 column vector of 1s and **I** is the *m*×*m* identity matrix. Then the desired correlation structure **A** is a block diagonal matrix of dimension *n*×*n* with ***τ***_1_ on the diagonal blocks and ***τ***_2_ on the off-diagonal blocks.

To achieve this structure for a binary random vector, first generate a realization **Y** from the multivariate normal distribution of dimension *n* with mean equal to the zero vector and variance–covariance matrix equal to the above-described correlation matrix **A**. Each component of the multivariate normal realization **Y** (*Y*_*i*_, *i*=1,…,*n*) is marginally distributed as a normal random variable with mean 0 and unit variance, and the correlation between any two components *Y*_*i*_ and *Y*_*j*_ is given by the (*i*,*j*)th entry of **A**.

To attain the binary sample with the desired correlation structure, let

where Φ(·) denotes the cumulative distribution function of the standard normal distribution and *P* is chosen to reflect the prevalence of malnutrition (*P*=*P*_0_ when simulating under the null hypothesis and *P*=*P*_a_ when simulating under the alternative hypothesis). Then *D*_*i*_ is a Bernoulli random variable with mean *P*{*Y*_*i*_≤Φ^−1^(*P*)}=*P* for *i*=1,…,*n* and **D**=(*D*_1_,*D*_2_,…,*D*_*n*_) is the resulting correlated sample of binary outcomes.

The correlation between any two of the resulting binary components *D* and *D*^′^ is given by

(1)

where *f*(·) is the probability density function of a bivariate normal distribution with mean **0** and variance–covariance matrix given by **I**(1−*τ*)+**11**^T^*τ*, where **I** is the 2×2 identity matrix and **1** is a 2×1 vector of 1s. The goal is to choose *τ* and *P* such that the resulting correlation is a specific value *ρ*. Define the function *ρ*(*P*,*τ*)≡corr(*D*,*D*^′^). Fig. 1 plots *ρ*(*P*,*τ*) against *τ* for a range of *P*. Although no closed form solution to the double integral exists in equation (1), numerical integration yields highly precise approximations and can be implemented in many software packages. Here, numerical integration was performed by using the library in R version 2.6.0.

This approximation is used to simulate binary outcomes with correlation *ρ* and mean *P*. For example, to simulate binary outcomes with correlation *ρ*=0.05 and mean *P*=0.10 requires simulation in the multivariate normal with correlation *τ*=0.131. Table 7 outlines the values of *τ* that were used to simulate binary outcomes with correlation *ρ* and mean *P* in this study.

**Table 7:** Values of *τ* needed to simulate binary outcomes with correlation *ρ* and mean *P*

*Correlation ρ*	*Values of τ for the following prevalences P:*			
	*0.05*	*0.10*	*0.15*	*0.20*
0.05	0.177	0.131	0.110	0.098
0.10	0.305	0.242	0.210	0.191
0.15	0.407	0.339	0.301	0.277
0.20	0.493	0.424	0.385	0.359
0.25	0.568	0.501	0.462	0.435
